# Real-world clinical outcome of unresectable locally advanced & de-novo metastatic pancreatic ductal adenocarcinoma: a multicentre retrospective study

**DOI:** 10.1186/s12885-024-13386-0

**Published:** 2025-01-03

**Authors:** Mohamed Aseafan, Ali H. Alfakeeh, Emad Tashkandi, Mervat Mahrous, Mohammed Alghamdi, Bader Alshamsan, Marwan Al-Hajeili, Safwan Bakhsh, Kanan Alshammari, Fahad A. Almugbel, Abdulhameed H. Alfagih, Ahmed Allehebi, Mohamed Montaser, Mohamed Hamdy Elsafty, Khaled Abd Elaziz Elnaghi, Ibrahim Issa, Eesa Bakshi, Sadeem AlSubaie, Bandar AlMutairi, Hoda Mokhtar, Mohamed Aboelatta, Nedal Bukhari, Ali M. Alzahrani, Tusneem Elhassan, Ali Alqahtani, Shouki Bazarbashi

**Affiliations:** 1 Section of Medical Oncology, Department of Internal Medicine, Security Forces Hospital, Riyadh, Saudi Arabia; 2 Comprehensive Cancer Center, Medical Oncology, King Fahad Medical City, Riyadh, Saudi Arabia; 3 College of Medicine, Umm Al-Qura University, Makkah, Saudi Arabia; 4 Department of Oncology, Prince Sultan Military Medical City, Riyadh, Saudi Arabia; 5 Collage of Medicine, Minia University, Minia, Egypt; 6 Oncology Center, King Saud University Medical City, Riyadh, Saudi Arabia; 7 Department of Medicine, College of Medicine, Qassim University, Buraydah, Saudi Arabia; 8 Prince Faisal Cancer Center, King Fahad Specialist Hospital, Qassim Health Clusster, Buraydah, Saudi Arabia; 9 Department of Medicine, King Abdulaziz University, Jeddah, Kingdom of Saudi Arabia; 10 Department of Medical Oncology, King Faisal Specialist Hospital and Research Center-Jeddah, Jeddah, Saudi Arabia; 11 Department of Medical Oncology, Ministry of National Guard Health Affairs, Riyadh, Saudi Arabia; 12 Department of Medical Oncology, Cancer Centre of Excellence, King Faisal Specialist Hospital and Research Centre, Riyadh, Saudi Arabia; 13 Faculty of Medicine, Ain Shams University, Cairo, Egypt; 14 Medical Oncology, NCI Cairo University, Cairo, Egypt; 15 Oncology Centre, Faculty of Medicine, Mansoura University, Mansoura, Egypt; 16 College of Medicine, Alfaisal University, Riyadh, Saudi Arabia; 17 Pathology and Laboratory Medicine, Security Forces Hospital, Riyadh, Saudi Arabia; 18 Research Unit, Cancer Centre of Excellence, King Faisal Specialist Hospital and Research Centre, Riyadh, Saudi Arabia

**Keywords:** Pancreatic cancer, Ductal adenocarcinoma, Locally advance, De-novo, Survival, Saudi Arabia

## Abstract

**Background:**

Pancreatic ductal adenocarcinoma (PDAC) remains one of the most lethal malignancies, with limited treatment options yielding poor outcomes. This study aimed to evaluate the real-world clinical characteristics, treatment patterns, and outcomes of patients with locally advanced unresectable and de-novo metastatic PDAC in Saudi Arabia, providing regional data to compare with international benchmarks.

**Methods:**

This is a retrospective, multicentre study involving 350 patients diagnosed with unresectable locally advanced or de-novo metastatic PDAC between January 2015 and November 2023. Data were collected from 10 oncology centers across Saudi Arabia.

**Results:**

The median age at diagnosis was 60 years, with 63% of patients presenting with multiple metastatic sites, primarily in the liver (66.3%). FOLFIRINOX was the most common first-line treatment (55.1%), followed by gemcitabine plus nab-paclitaxel (15.1%). The median PFS for first-line treatment was 5.3 months, with FOLFIRINOX achieving the longest PFS (6.5 months). The median OS was 10.34 months for the entire cohort, with better survival outcomes observed in patients receiving FOLFIRINOX (12.3 months). Independent prognostic factors for PFS and OS included performance status, first-line regimen, and neutrophil-lymphocyte ratio (NLR). Among patients tested, 7.1% had deficient mismatch repair (d-MMR), and 5.8% harbored BRCA mutations.

**Conclusions:**

This real-world study confirms that clinical outcomes for locally advanced unresectable and metastatic PDAC in Saudi Arabia are consistent with international data, with FOLFIRINOX showing superior outcomes over gemcitabine-based regimens. However, both treatments reflect the persistent poor prognosis of PDAC, underscoring the need for novel therapeutic strategies. Further research is warranted to optimize treatment selection and improve survival outcomes in this population.

## Background

Pancreatic ductal adenocarcinoma (PDAC) is a fatal disease with raising incidence and mortality accounting for the third leading cause of cancer-related deaths in USA and the seventh leading cause of cancer related deaths worldwide [1, 2]. PDAC is projected to be the second leading cause of cancer-related mortality by 2030 [3, 4]. Majority of patients present in a late stage making surgical resection the only curative treatment not feasible [5]. Chemotherapy continues to be the main stay of treatment for patients with locally advanced unresectable disease and metastatic disease at diagnosis and despite recent advances in management the five-year survival rate remains suboptimal with less than 13% and still lack an effective screening program [6, 7].

The current standard of care first-line treatment regimen for locally advance (LA) unresectable and metastatic PDAC includes: oxaliplatin, irinotecan, fluorouracil, and leucovorin (FOLFIRINOX), liposomal irinotecan, oxaliplatin, leucovorin, and fluorouracil (NALIRIFOX), gemcitabine plus nab-paclitaxel (GnP), and single agent gemcitabine (Gem) [8–11]. FOLFIRNOX and GnP have demonstrated overall survival benefit over Gem as single agent making both options standard of care with no comparative study answering which regimen is better [9, 10]. Recently, NALIRIFOX was tested against GnP showing superiority [11].

There is less consensus when it comes to second line treatment options as most studied proven efficacious therapies were after progression on gemcitabine-based therapy with limited data on options post FOLIRINOX as first line treatment [12–15]. Second-line GnP following progression on FOLFIRINOX have demonstrated a 6-month overall survival (OS) rate of 72.5% compared to only 20% in patients receiving best supportive care (BSC) [16]. Data about pancreatic cancer in our part of the world is scares. Hence, we aim to assess our patient characteristic and treatments trends and outcomes.

## Methods

This is a multicentre retrospective observational study to evaluate patient characteristic, treatment trends, and clinical outcome for LA unresectable and de-novo metastatic PDAC. Medical records of patients diagnosed PDAC treated in 10 centres around Saudi Arabia were retrospectively reviewed. Patients who were 18-years of age or older and diagnosed from January 1st, 2015, to November 1st, 2023, with LA unresectable or de-novo metastatic PDAC with no prior pancreatic surgery and received palliative first-line systemic therapy were included. Patients with prior malignancy, or received prior chemotherapy were excluded.

This study was conducted in accordance with the principles of the Declaration of Helsinki [17]. Approval was granted by the institutional review board at Security Forces Hospital research #23-645-09 on 22 June 2023. Waiver of consent was granted by the institutional review board given the retrospective nature of the study.

### Statistical analysis

Patient characteristics were summarized using frequencies and percentages for categorical variables and medians and ranges for continuous variables.

Continuous variables requiring categorization were classified using optimal cutoff values derived from Receiver Operating Characteristic (ROC) curve analysis. The optimal cutoffs were determined by maximizing the sum of sensitivity and specificity.

Progression-free survival-1 (PFS-1) was calculated as the time from the start of first-line therapy to either progression or death. PFS-2 was calculated as the time of start second-line therapy to either progression or death. OS was calculated as the time from the start of first-line therapy to the date of death or last follow-up.

Survival analysis was conducted using the Kaplan-Meier method to estimate survival functions, with survival probabilities computed at each time point. Greenwood’s formula was applied to determine the variance of survival estimates and calculating confidence intervals for the survival probabilities. Censoring was implemented for subjects who did not experience the event of interest during the study period or who were lost to follow-up.

Kaplan-Meier survival curves were used to visualize differences in survival between study groups and statistical comparisons between survival distributions were assessed using the log-rank test.

To assess the independent impact of risk factors on survival, a Cox proportional hazards regression model was employed. Prior to model development, the proportional hazards assumption was evaluated using Schoenfeld residuals to verify that the effect of covariates on the hazard rate remained constant over time. If this assumption was violated, the covariate was incorporated as a time-dependent variable. Interaction terms between the first line regimen and significant covariates were examined to detect potential effect modification.

The final multivariate model was constructed using a backward elimination approach, where all candidate covariates were initially included in the model. Covariates were sequentially removed based on the likelihood ratio test, with a criterion of p-value > 0.10 for exclusion from the model. Hazard ratios and their corresponding 95% confidence intervals were calculated for the covariates that remained in the final model to quantify their association with the risk of the event. All multivariate regression models were developed with adjustments for the center effect. Statistical analyses were performed using STATA/BE 17, with the significance threshold set at a two-tailed alpha level of 0.05.

## Results

Three hundred fifty patients were enrolled from 10 cancer centers in Saudi Arabia, all diagnosed with either de novo metastatic or locally advanced unresectable PDAC. Among them, 255 (73%) had de novo metastatic PDAC. The median age at diagnosis was 60 years (IQR 53–66). The most common primary site was the head of the pancreas, accounting for 205 patients (58.6%), followed by the body in 115 patients (32.9%). The median number of metastatic sites was 2 (IQR 1–3), and 63% of patients presented with more than one metastatic site at diagnosis. The liver was the most frequent site of metastasis, observed in 66.3% of cases, followed by lymph nodes (28%) and lungs (25.1%). Carbohydrate antigen 19 − 9 (CA19-9) levels were elevated in 83.4% of patients. Among those tested for mismatch repair (MMR) status (85 patients), six (7.1%) had deficient MMR (d-MMR). BRCA testing was conducted in 69 patients, revealing BRCA mutations in four individuals (5.8%), including three with BRCA1 mutations and one with BRCA2 mutation. Detailed clinicopathological characteristics of the patients are summarized in Table 1.

**Table 1:** Patient characteristics of patients diagnosed with locally advanced unresectable or metastatic pancreatic ductal adenocarcinoma (*n* = 350)

Variables	All patients 350 *N* (%)
Age (years)	
<65	247 (70.5)
≥65	103 (29.5)
Sex	
Female	129 (37)
Male	221 (63)
Stage at diagnosis	
Locally advance unresectable	95 (27)
Metastatic	255 (73)
Comorbidities	
Diabetes	199 (57)
Hypertension	155 (44)
Coronary artery disease	23 (7)
Renal impairment	13 (4)
The primary site of the pancreas	
Head	205 (58.6)
Body	115 (32.9)
Tail	57 (6.3)
Unknown	22 (2.2)
Pathology differentiation	
Well-differentiated	15 (4.3)
Moderately differentiated	164 (46.9)
Poorly differentiated	57 (16.3)
Unknown	114 (32.5)
ECOG-Performance status	
0–1	196 (56)
2	133 (38)
3	9 (2.6)
Unknown	12 (3.4)
Site of metastasis	
Liver	232 (66.3)
Lung	88 (25.1)
Bone	30 (8.6)
Peritoneum	76 (21.7)
Lymph nodes	98 (28)

The most common first-line treatment was FOLFIRINOX, administered to 193 patients (55.1%), followed by Gemcitabine + Nab-paclitaxel in 53 patients (15.1%). The median number of cycles for the entire group was six, with specific medians of 8, 6, and 4 cycles for FOLFIRINOX, Gemcitabine + Nab-paclitaxel, and Gemcitabine single agent, respectively. 60% of patient needed dose reduction in the first-line treatment. Approximately 49% of the cohort received second-line treatment, with GnP being the most common regimen (35.5%), followed by Gemcitabine single agent (22.7%). The median number of cycles in the second line was 4 (IQR 3-7.75), similar across all regimens. Thirty-five patients (10%) received third-line therapy. Among locally advanced unresectable patients, 36% received local radiotherapy, with concurrent chemoradiotherapy (CCRT) and stereotactic body radiotherapy (SBRT) being utilized in 41.2% and 29.4% of cases, respectively. Table 2 details the treatment modalities used in managing the cohort. Biliary stenting was performed in 184 patients (52.6%), and 46.3% developed deep vein thrombosis (DVT) or pulmonary embolism (PE).

**Table 2:** Treatment regimens and clinical outcomes of patients with unresectable locally advanced and de-novo pancreatic ductal adenocarcinoma

Variables	*N* (%)
First-line treatment regimen	
FOLFIRINOX	193 (55.1)
Gemcitabine + Nab-paclitaxel	53 (15.1)
Gemcitabine + Capecitabine	8 (2.3)
Gemcitabine	74 (21.1)
Others	22 (6.3)
Maintenance therapy	
Yes	46 (13.1)
No	294 (84)
Reasons for stopping chemotherapy	
Disease progression	216 (61.7)
Completed 6 months of therapy	40 (11.4)
Toxicity	23 (6.6)
Patient request	14 (4)
Others	57 (16.2)
Received 2nd line therapy	172 (49)
Gemcitabine-nabpaclitaxel	61 (35.5%)
Gemcitabine	39 (22.7)
FOLFOX	25 (14.5)
FOLFIRI	9 (5.2)
Others	38 (22.1)
Received 3rd line therapy	35 (10)
Received Local radiotherapy	34 (36.6)
Types of Radiotherapy received	
CCRT	14 (41.2)
Palliative radiotherapy	9 (26.5)
SBRT	10 (29.4)

The median time to first response evaluation with CT/MRI was 10 weeks (IQR 8–14 weeks), with imaging performed for response evaluation in 324 patients (92.6%). The objective response rate (ORR) for first-line treatment was 31.1%, with the highest rates observed in FOLFIRINOX (38.3%), followed by GnP (34.4%), and Gemcitabine single agent (9.5%). Among 172 patients receiving second-line treatment, 37.8% achieved an ORR, with all responses being Partial Response (PR). The PR rates were 55.6% for FOLFIRI, 41% for GnP, 36% for FOLFOX, and 33.3% for Gemcitabine.

**Fig. 1:**
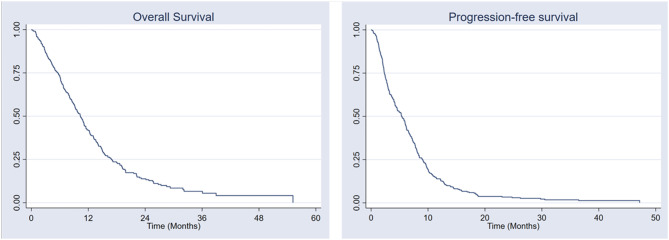
Progression-free and overall survival for unresectable locally-advanced and de-novo metastatic pancreatic ductal adenocarcinoma for all patients

The median follow-up period for our study was 28 (95%.CI: 25, 40) months. The median PFS-1 for the whole group was 5.3 months (95% CI 4.48–6.16). The median PFS-1 for FOLFORINOX, GnP, and Gem were 6.5 months, 6.2 months, and 2.5 months, respectively (P = ≤ 0.001). At time data was captured for analysis 9.4% of patients were still progression-free on first-line therapy. The median PFS-2 was 3.25 months (95% CI 2.84–3.66), with PFS-2 for GnP, FOLFIRI, and Gemcitabine at 3.8 months, 3.1 months, and 2.6 months, respectively (*p* = 0.202). The median OS was 10.34 months (95% CI 9.32–11.37). Figure 1 illustrates PFS-1 and OS for all patients. Univariate and multivariant analysis for PFS and OS is presented in Tables 3 and 4. Multivariate analysis showed that first-line treatment regimen, Eastern Cooperative Oncology Group performance status (ECOG-PS), NLR, and pathology grade were independent prognostic factors for PFS, while first-line treatment regimen type, ECOG-PS, and NLR were independent prognostic factors for OS.

**Table 3:** Median PFS and OS stratified by variables associated with advance pancreatic ductal adenocarcinoma

	mPFS (months)	95% Confidence interval (CI)	*P*-value	mOS (months)	95% CI	*P*-value
All patients	5.3	4.5–6.2		10.3	9.3–11.4	
Age			0.833			0.490
<65	5.5	4.5–6.4		10.6	9.4–11.8	
≥65	5.1	3.0-7.1		9.4	6.7–12.1	
Sex			0.699			0.703
Female	6	5.2–6.8		10.3	8.4–11.3	
Male	5.1	4.1-6.0		10.3	9.2–11.5	
Stage at presentation			0.410			0.207
Locally advance unresectable	4.9	2.8–7.1		11.5	8.9–14.0	
De-novo metastatic	5.5	4.6–6.4		9.9	8.6–11.1	
Tumor grade			0.034			0.113
Well-differentiated	8.1	3-13.1		16.1	7.0-25.3	
Moderately differentiated	5.8	3.1–6.4		10.8	9.2–12.4	
Poorly differentiated	3.7	2.4–5.1		9.2	7.3–11.2	
Metastatic site						
Liver			≤ 0.001			0.002
No	7	5.4–8.5		13.1	10.7–15.4	
Yes	4.6	3.8–5.4		9.2	7.9–10.5	
Lung			0.899			0.712
No	5.3	4.2–6.3		10.3	9.1–11.4	
Yes	6.2	5.1–7.3		10.8	8.4–13.2	
Peritoneum			0.451			0.964
No	5.3	4.4–6.2		10.6	9.4–11.8	
Yes	6	4–8		9.6	7.6–11.6	
Lymph nodes			0.605			0.808
No	5.1	4-6.1		10.3	9.0-11.7	
Yes	6.1	5-7.2		10.6	9.1–12.1	
ECOG-PS			≤ 0.001			≤ 0.001
0–1	6.2	5.3-7		12.4	10.7–14.2	
2	4.4	3.4–5.4		8.1	6.2–10.0	
3	1.9	0.3–3.4		2.9	0.8-5.0	
Albumin			0.026			0.708
< 35	5	3.8–6.1		10.4	9.0-11.8	
≥ 35	6.2	5.1–7.4		10.3	8.9–11.6	
Bilirubin			0.192			0.045
≤21	5.5	4.3–6.6		10.6	9.4–11.9	
>21	5.3	4-6.6		9.2	7.1–11.3	
CA 19 − 9			0.783			0.093
≤37	4.7	1.4–8.1		13.2	8.9–17.5	
>37	5.8	5.0-6.6		10.3	9.2–11.4	
Neutrophil-Lymphocyte-ratio (NLR)			0.007			0.011
≤1.97	6.6	5.8–7.4		11.8	10.0-13.6	
>1.97	4	3.2–4.8		8.9	7.6–10.2	
Platelet-Lymphocyte-ratio (PLR)			0.3			0.595
≤133.75	6.2	5.2–7.2		10.8	9.5–12.1	
>133.75	4.6	3.4–5.7		9.9	8.0-11.7	
First-line treatment			≤ 0.001			≤ 0.001
FOLFIRINOX	6.5	5.8–7.3		12.3	10.8–13.8	
Gemcitabine + Nab-paclitaxel	6.2	5.1–7.2		9.7	6.7–12.6	
Gemcitabine	2.5	1.9–3.1		5.2	3.4-7.0	

**Table 4:** Independent prognostic factor for PFS & OS by multi-variate analysis

Risk factors	Progression free survival			Overall Survival		
	HR	95%, CI	*p*-value	HR	95%, CI	*p*-value
First Line regimen			0.001			0.081
FILFIRINOX	1			1		
Gem/Nab-paclitaxel	1.26	0.8–1.9	0.259	1.06	0.7–1.5	0.751
Gemcitabine	2.22	1.5–3.3	< 0.001	1.51	1.1–2.2	0.033
Others	1.20	0.6–2.4	0.613	1.47	0.9–2.5	0.141
ECOG-PS			0.025			< 0.001
0–1	1			1		
2	1.11	0.8–1.5	0.533	1.50	1.1-2	0.011
3	3.89	1.5–10.4	0.007	4.55	2-10.2	< 0.001
Neutrophil-Lymphocyte-ratio			0.014			< 0.021
<=1.97	1			1		
>1.97	1.438	1.1–1.9		1.34	1-1.7	
Pathology Grade			0.031			n/a
Moderately differentiated	1					
Poorly differentiated	1.40	0.9-2	0.059			
Well differentiated	0.62	0.3–1.1	0.101			

## Discussion

Our study serves to characterize patients’ characteristics, treatment trends, and outcomes of LA unresectable and metastatic PDAC in our region and compare our treatment outcome with available literature. Data concerning this disease is scarce in our region that makes this study of importance.

The median age at diagnosis in our population was 60 years that is younger than reports from western populations and comparable to reports from Asia and North Africa with exception of Japan [18–27]. This can be explained by the relatively younger population in these countries in contrary to western population and Japan. PDAC has a male predominance, and the most common site is the head of pancreas in most reports similar to our study [18, 19, 24, 25, 27, 28].

Real-world outcomes of cancer treatment against prospective randomized control trials (RCTs) can help as a benchmark to identify gaps to deliver best practice, figure current clinical challenges, and optimize the use of available treatments [29]. Outcomes of treated advance PDAC for the whole group in our study was comparable to reports from other parts of the World [27, 30, 31].

Majority of real-world retrospective reports comparing the efficacy of FOLFIRINOX with GnP as a first line have resulted in better ORR, and longer PFS and OS in favour of FOLFIRINOX which is similar to our patient population [23, 25, 28, 30–33]. On the other hand, Prospective studies comparing the efficacy of FOLFIRINOX with GnP as a first-line are scarce with one trial from Japan (JCOG1611, GENERATE) that failed to show superiority of FOLFIRINOX over GnP, in contrast it resulted in numerically longer OS in GnP arm and was terminated for futility [34]. Recently, the NAPOLI 3 trail compared NALIRIFOX vs. GnP in the first-line setting of patients with mPDAC. The trial showed superiority of NALIRIFOX over GnP with median OS of 11·1 months vs. 9·2 months (HR 0·83; 95% CI 0·70–0·99; *p* = 0·036), adding another option to the first-line treatment landscape [11]. Currently, there are no studies comparing FOLFIRINOX with NALIRIFOX. Since NALIRIFOX was not approved during our study period we believe that our patient cohort represent a homogeneously treated patients with standard of care.

Patients who received FOLFIRINOX as first-line treatment in our study have achieved an ORR of 38.3%, median PFS was 6.5 months (95% CI, 5.8–7.3; p = ≤ 0.001), and median OS was 12.3 months (95% CI, 10.8–13.8; p = ≤ 0.001) that was comparable to what was reported in PRODIGE 4/ACCORD 11 trial showing 31.6% ORR, median PFS and OS was 6.4 months, and 11.1 months, respectively [9]. Similarly, the cohort who received GnP regimen as first-line treatment yielded an ORR of 34.4%, median PFS and OS was 6.2 months, and 9.7 months which was comparable to the reported results of MPACT trial showing a 23% ORR, 5.5 months, and 8.5 months median PFS and OS, respectively [10]. Single-agent Gem as first-line in our study have resulted in 9.5% ORR, mPFS was 2.5 months, and mOS was 5.2 months. This is secondary to selecting Gem in older and less fit patients unlike randomised controlled trials [8–10].

In our cohort 60% of patient receiving first-line treatment required any dose reduction with FOLFIRINOX having the highest percentage (70%), 69% in GnP, and 33% in Gem. Our study did not assess for the trend in using standard vs. modified FOLFIRINOX. This can explain the high rate of dose reduction in FOLFIRINOX regimen. It is noteworthy that dose reduction in FOLFIRINOX regimen does not alter its efficacy as shown in a recent meta-analysis of modified FOLFIRINOX vs. standard FOLFIRINOX in metastatic PDAC [35]. In this meta-analysis the ORR for was (33.8% *versus* 28.2%; *p* = 0.1) for modified FOLFIRINOX, and standard FOLFIRINOX, respectively. This finding is comparable with the ORR of 31.6% in the PRODIGE 4/ACCORD 11 trial [9, 35]. In addition, the rates of neutropenia and febrile neutropenia in the modified FOLFIRINOX group were lower as reported in the PRODIGE 24/ACCORD 24 trial [36].

49% of our patients have received second-line treatment similar to reported literature [13, 21, 25]. In our study gemcitabine-based treatment was the most common used representing 58.2% which is in line with FOLFIRINOX being the most common first-line regimen. Most studies in the second line setting have evaluated 5-FU based regimens. Second-line GnP post FOLFIRINOX was studied in single arm phase II trial resulting in median PFS of 5.8 months (95% CI, 4.3–8.7) which is higher than our cohort with median PFS was 3.8 months [16].

Our multi-variate analysis has showed no difference between FOLFIRINOX and GnP when used as first-line regimen, however both were better than Gem single agent. Additionally, ECOG-PS, and NLR were independent prognostic factors for both PFS and OS that is consistent with other reports in the literature [24, 25, 32]. Other reports have highlighted that age and CA 19 − 9 as prognostic factor which we did not appreciate in our analysis [25, 26, 37]. Some reports have described site of metastasis as a prognostic factor with liver metastasis having worse prognosis, in our study liver metastasis was not an independent prognostic factor [25, 26, 37]. Patients with unresectable LA PDAC had numerically better PFS and OS compared with de novo metastatic disease, but this did not reach statistical significance possibly due to the small number of patients.

While our study demonstrates comparable outcomes to global benchmarks, it is important to consider the unique context of the Saudi Arabian healthcare system. For example, while FOLFIRINOX and gemcitabine-based regimens are considered standard of care globally, treatment accessibility and physician preferences may differ across regions. For instance, modified FOLFIRINOX is frequently used in Western settings to improve tolerability, whereas data on its utilization in Saudi Arabia remain limited due to variability in treatment protocols across centers. Additionally, variations in patient demographics and risk factors, such as obesity or diabetes, could contribute to observed differences in survival rates compared to Asian cohorts. Furthermore, healthcare infrastructure in Saudi Arabia, though advanced, may face unique challenges such as centralized oncology services and limited access to molecular testing, as seen in the low rates of BRCA and mismatch repair testing in our cohort (5.8% and 7.1%, respectively). In contrast, countries with well-established precision oncology programs report higher rates of genetic profiling, which may allow for more personalized treatment strategies. These differences underscore the importance of regional studies like ours, as they provide valuable insights into the real-world application of global treatment guidelines and highlight areas for improvement in healthcare delivery and accessibility.

Our manuscript highlights the outcomes of patients with locally advanced unresectable and metastatic pancreatic ductal adenocarcinoma treated in Saudi Arabia, emphasizing the need for future research in several key areas. Future studies should explore molecular biomarkers to identify genetic alterations that could guide treatment decisions, integrate novel therapeutic agents such as targeted therapies and immunotherapies to improve patient outcomes, and assess patient-reported outcomes to better understand the impact of treatments on quality of life. Additionally, focusing on personalized treatment strategies and long-term follow-up could enhance understanding and management of this aggressive cancer, ultimately aiming to improve survival rates and treatment effectiveness in this patient population.

Our study has several limitations, First, being retrospective, second, the choice of first‑line treatment regimen was done by the treating physicians from different centers with no clear criteria have been established and possibly influenced by treatment availability. Third, the protocol used of each regimen differs for each center. Lastly, some prognostic factors, such as body mass index (BMI), smoking, and molecular testing were not examined due to lack of data. On the other hand, the strength of our study lies in its large sample size and its multicenter design, which includes ten major centers representing diverse geographical regions of the country in a real-world setting.

## Conclusions

In conclusion, our real-world data have demonstrated that our current practice across Saudi Arabia centers for treating advance PDAC matches the global standards with similar outcomes observed in the pivotal RCT. First-line treatment regimen choice is an independent prognostic factor and still we have no clear answer if FOLFIRINOX is superior to GnP. Hence, our findings provide important information about our patient’s clinical characteristics, guide policy makers in future initiatives, local guidelines, and clinical decision‑making.

## Data Availability

All data and documents needed will be provided upon request through email: mjaseafan@gmail.com.

## Abbreviations

PDAC: Pancreatic ductal adenocarcinoma
NLR: Neutrophil-lymphocyte ratio
LA: Locally advance
FOLFIRINOX: Oxaliplatin, irinotecan, fluorouracil, and leucovorin
NALIRIFOX: Liposomal irinotecan, oxaliplatin, leucovorin, and fluorouracil
GnP: Gemcitabine plus nab-paclitaxel
Gem: Gemcitabine
BSC: Best supportive care
OS: Overall survival
PFS-1: Progression-free survival-1
CA19-9: Carbohydrate antigen 19 − 9
MMR: Mismatch repair
d-MMR: Deficient MMR
CCRT: Concurrent Chemoradiotherapy
SBRT: Stereotactic Body Radiotherapy
DVT: Deep Vein Thrombosis
PE: Pulmonary Embolism
ECOG-PS: Eastern Cooperative Oncology Group performance status
BMI: Body mass index

## References

[1] Siegel RL, Miller KD, Jemal A. Cancer statistics, 2020. CA Cancer J Clin. 2020;70:7–30. 10.3322/caac.21590. 31912902 10.3322/caac.21590

[2] Sung H, Ferlay J, Siegel RL, et al. Global Cancer statistics 2020: GLOBOCAN estimates of incidence and Mortality Worldwide for 36 cancers in 185 countries. CA Cancer J Clin. 2021;71:209–49. 10.3322/caac.21660. 33538338 10.3322/caac.21660

[3] Huang J, Lok V, Ngai CH, et al. Worldwide Burden of, risk factors for, and trends in Pancreatic Cancer. Gastroenterology. 2021;160:744–54. 10.1053/j.gastro.2020.10.007. 33058868 10.1053/j.gastro.2020.10.007

[4] Rahib L, Wehner MR, Matrisian LM, Nead KT. Estimated projection of US Cancer incidence and death to 2040. JAMA Netw Open. 2021;4:e214708. 10.1001/jamanetworkopen.2021.4708. 33825840 10.1001/jamanetworkopen.2021.4708 PMC8027914

[5] Cancer Stat Facts. Pancreatic Cancer.

[6] Kenner BJ, Chari ST, Maitra A, et al. Early detection of pancreatic Cancer-a defined future using lessons from other cancers: a White Paper. Pancreas. 2016;45:1073–9. 10.1097/MPA.0000000000000701. 27518362 10.1097/MPA.0000000000000701 PMC4993121

[7] Mizrahi JD, Surana R, Valle JW, Shroff RT. Pancreatic cancer. Lancet. 2020;395:2008–20. 10.1016/S0140-6736(20)30974-0. 32593337 10.1016/S0140-6736(20)30974-0

[8] Burris HA 3rd, Moore MJ, Andersen J, et al. Improvements in survival and clinical benefit with gemcitabine as first-line therapy for patients with advanced pancreas cancer: a randomized trial. J Clin Oncol. 1997;15:2403–13. 10.1200/JCO.1997.15.6.2403. 10.1200/JCO.1997.15.6.2403 9196156

[9] Conroy T, Desseigne F, Ychou M, et al. FOLFIRINOX versus gemcitabine for metastatic pancreatic cancer. N Engl J Med. 2011;364:1817–25. 10.1056/NEJMoa1011923. 21561347 10.1056/NEJMoa1011923

[10] Von Hoff DD, Ervin T, Arena FP, et al. Increased survival in pancreatic cancer with nab-paclitaxel plus gemcitabine. N Engl J Med. 2013;369:1691–703. 10.1056/NEJMoa1304369. 24131140 10.1056/NEJMoa1304369 PMC4631139

[11] Wainberg ZA, Melisi D, Macarulla T, et al. NALIRIFOX versus nab-paclitaxel and gemcitabine in treatment-naive patients with metastatic pancreatic ductal adenocarcinoma (NAPOLI 3): a randomised, open-label, phase 3 trial. Lancet. 2023;402:1272–81. 10.1016/S0140-6736(23)01366-1. 37708904 10.1016/S0140-6736(23)01366-1 PMC11664154

[12] Wang-Gillam A, Li CP, Bodoky G, et al. Nanoliposomal irinotecan with fluorouracil and folinic acid in metastatic pancreatic cancer after previous gemcitabine-based therapy (NAPOLI-1): a global, randomised, open-label, phase 3 trial. Lancet. 2016;387:545–57. 10.1016/S0140-6736(15)00986-1. 26615328 10.1016/S0140-6736(15)00986-1

[13] Yoo C, Hwang JY, Kim JE, et al. A randomised phase II study of modified FOLFIRI.3 vs modified FOLFOX as second-line therapy in patients with gemcitabine-refractory advanced pancreatic cancer. Br J Cancer. 2009;101:1658–63. 10.1038/sj.bjc.6605374. 19826418 10.1038/sj.bjc.6605374 PMC2778540

[14] Zaanan A, Trouilloud I, Markoutsaki T, et al. FOLFOX as second-line chemotherapy in patients with pretreated metastatic pancreatic cancer from the FIRGEM study. BMC Cancer. 2014;14:441. 10.1186/1471-2407-14-441. 24929865 10.1186/1471-2407-14-441 PMC4075567

[15] Zaniboni A, Aitini E, Barni S, et al. FOLFIRI as second-line chemotherapy for advanced pancreatic cancer: a GISCAD multicenter phase II study. Cancer Chemother Pharmacol. 2012;69:1641–5. 10.1007/s00280-012-1875-1. 22576338 10.1007/s00280-012-1875-1

[16] Huh G, Lee HS, Choi JH, et al. Gemcitabine plus Nab-Paclitaxel as a second-line treatment following FOLFIRINOX failure in advanced pancreatic cancer: a multicenter, single-arm, open-label, phase 2 trial. Ther Adv Med Oncol. 2021;13:17588359211056179. 10.1177/17588359211056179. 34790261 10.1177/17588359211056179 PMC8591648

[17] World Medical A. World Medical Association Declaration of Helsinki: ethical principles for medical research involving human subjects. JAMA. 2013;310:2191–4. 10.1001/jama.2013.281053. 24141714 10.1001/jama.2013.281053

[18] Body A, Wong R, Shapiro J, et al. Use and outcomes of chemotherapy for metastatic pancreatic cancer in Australia. Intern Med J. 2022;52:49–56. 10.1111/imj.15094. 33040452 10.1111/imj.15094

[19] Eddfair MM, Abdulrahman O, Alqawi O, et al. Correlations of demographical and clinicopathological features with patient outcome of pancreatic ductal adenocarcinoma: a retrospective study (2010–2018) from a Libyan cohort. J Cancer Res Ther. 2023;19:745–52. 10.4103/jcrt.jcrt_1778_21. 37470604 10.4103/jcrt.jcrt_1778_21

[20] Elias R, Cockrum P, Surinach A, Wang S, Chul Chu B, Shahrokni A. Real-world impact of age at diagnosis on treatment patterns and survival outcomes of patients with metastatic pancreatic ductal adenocarcinoma. Oncologist. 2022;27:469–75. 10.1093/oncolo/oyac028. 35278079 10.1093/oncolo/oyac028 PMC9177118

[21] King G, Ittershagen S, He L, Shen Y, Li F, Villacorta R. Treatment patterns in US patients receiving first-line and second-line therapy for metastatic pancreatic ductal adenocarcinoma in the Real World. Adv Ther. 2022;39:5433–52. 10.1007/s12325-022-02317-9. 36197644 10.1007/s12325-022-02317-9 PMC9618512

[22] Kordes M, Yu J, Malgerud O, Gustafsson Liljefors M, Lohr J. Survival benefits of chemotherapy for patients with Advanced Pancreatic Cancer in a clinical real-world cohort. Cancers (Basel). 2019;11:103390cancers11091326. 10.3390/cancers11091326 PMC6769947 31500236

[23] Pijnappel EN, Dijksterhuis WPM, van der Geest LG, et al. First- and second-line palliative systemic treatment outcomes in a real-world metastatic pancreatic Cancer cohort. J Natl Compr Canc Netw. 2021;20:443–e450443. 10.6004/jnccn.2021.7028. 34450595 10.6004/jnccn.2021.7028

[24] Shimoyama R, Imamura Y, Uryu K, et al. Real–world treatment outcomes among patients with metastatic pancreatic cancer in Japan: the Tokushukai real–world data project. Mol Clin Oncol. 2023;19:98. 10.3892/mco.2023.2694. 37953858 10.3892/mco.2023.2694 PMC10636700

[25] Taieb J, Seufferlein T, Reni M, et al. Treatment sequences and prognostic/predictive factors in metastatic pancreatic ductal adenocarcinoma: univariate and multivariate analyses of a real-world study in Europe. BMC Cancer. 2023;23:877. 10.1186/s12885-023-11377-1. 37723453 10.1186/s12885-023-11377-1 PMC10506331

[26] Wu L, Zhu L, Xu K, et al. Clinical significance of site-specific metastases in pancreatic cancer: a study based on both clinical trial and real-world data. J Cancer. 2021;12:1715–21. 10.7150/jca.50317. 33613759 10.7150/jca.50317 PMC7890328

[27] Yasmeen S, Arshad F, Shaukat S, Badar F, Kazmi SAS, Ahmad U. Efficacy of Chemotherapy for locally Advanced and metastatic pancreatic Cancer: a real-life experience and outcome from a Tertiary Care Centre. J Cancer Allied Spec. 2021;7:e409. 10.37029/jcas.v7i2.409. 37197218 10.37029/jcas.v7i2.409 PMC10166315

[28] Jooste V, Bengrine-Lefevre L, Manfredi S, et al. Management and outcomes of Pancreatic Cancer in French Real-World Clinical Practice. Cancers (Basel). 2022;14. 10.3390/cancers14071675. 10.3390/cancers14071675 PMC8996902 35406447

[29] Tang M, Pearson SA, Simes RJ, Chua BH. Harnessing real-world evidence to Advance Cancer Research. Curr Oncol. 2023;30:1844–59. 10.3390/curroncol30020143. 36826104 10.3390/curroncol30020143 PMC9955401

[30] Cartwright TH, Parisi M, Espirito JL, et al. Clinical outcomes with first-line chemotherapy in a large retrospective study of patients with metastatic pancreatic Cancer treated in a US Community Oncology setting. Drugs Real World Outcomes. 2018;5:149–59. 10.1007/s40801-018-0137-x. 29946913 10.1007/s40801-018-0137-x PMC6119168

[31] Chan KKW, Guo H, Cheng S, et al. Real-world outcomes of FOLFIRINOX vs gemcitabine and nab-paclitaxel in advanced pancreatic cancer: a population-based propensity score-weighted analysis. Cancer Med. 2020;9:160–9. 10.1002/cam4.2705. 31724340 10.1002/cam4.2705 PMC6943167

[32] Franco F, Camara JC, Martin-Valades JI, et al. Clinical outcomes of FOLFIRINOX and gemcitabine-nab paclitaxel for metastatic pancreatic cancer in the real world setting. Clin Transl Oncol. 2021;23:812–9. 10.1007/s12094-020-02473-w. 32857340 10.1007/s12094-020-02473-w

[33] Wang Y, Camateros P, Cheung WY. A real-world comparison of FOLFIRINOX, Gemcitabine Plus nab-Paclitaxel, and Gemcitabine in Advanced pancreatic cancers. J Gastrointest Cancer. 2019;50:62–8. 10.1007/s12029-017-0028-5. 29143916 10.1007/s12029-017-0028-5

[34] Ohba A, Ogawa MOG, Okusaka T, Kobayashi S, Yamashita T, Ikeda M, Yasuda I, Sugimori K, Sasahira N, Ikezawa K, Miki I, Okano N, Mizuno N, Furukawa M, Shirakawa H, Sano Y, Katayama H, Furuse J, Ueno M. Nab-paclitaxel plus gemcitabine versus modified FOLFIRINOX or S-IROX in metastatic or recurrent pancreatic cancer (JCOG1611, GENERATE): a multicentred, randomized, open-label, three-arm, phase II/III trial. Ann Oncol. 2023;34:S849. 10.1016/j.annonc.2023.09.2565.

[35] Jung K, Choi S, Song H, et al. Real-world dose reduction of standard and modified FOLFIRINOX in metastatic pancreatic cancer: a systematic review, evidence-mapping, and meta-analysis. Ther Adv Med Oncol. 2023;15:17588359231175441. 10.1177/17588359231175441. 37441327 10.1177/17588359231175441 PMC10333643

[36] Conroy T, Hammel P, Hebbar M, et al. FOLFIRINOX or Gemcitabine as Adjuvant Therapy for Pancreatic Cancer. N Engl J Med. 2018;379:2395–406. 10.1056/NEJMoa1809775. 30575490 10.1056/NEJMoa1809775

[37] AlGhamdi HJ, Alfaifi SA, Alolayan AA, Musaad SM, Jazieh AM. Pancreatic cancer in Saudi patients treated at tertiary institution. Ten years retrospective study. Saudi Med J. 2013;34:604–8. 23756925

